# A Germline-Encoded Structural Arginine Trap Underlies the Anti-DNA Reactivity of a Murine V Gene Segment

**DOI:** 10.3390/ijms22094541

**Published:** 2021-04-26

**Authors:** Ronny Petterson dos Santos Araújo, Renato Kaylan Alves França, Napoleão Fonseca Valadares, Andrea Queiroz Maranhão, Marcelo Macedo Brigido

**Affiliations:** 1 Department of Cellular Biology, Institute of Biology, University of Brasilia, Brasilia 70910-900, Brazil; ronnypetter@hotmail.com (R.P.d.S.A.); renatokaylan@gmail.com (R.K.A.F.); napo@unb.br (N.F.V.); andreaqm@unb.br (A.Q.M.); 2 Instituto Nacional de Ciência e Tecnologia (iii-INCT), São Paulo 05403-000, Brazil

**Keywords:** V genes, anti-DNA, pre-BCR, autoreactivity, B cell’s repertoire

## Abstract

Autoimmunity may have its origins of early repertoire selection in developmental B cells. Such a primary repertoire is probably shaped by selecting B cells that can efficiently perform productive signaling, stimulated by self-antigens in the bone marrow, such as DNA. In support of that idea, we previously found a V segment from V_H_10 family that can form antibodies that bind to DNA independent of CDR3 usage. In this paper we designed four antibody fragments in a novel single-chain pre-BCR (scpre-BCR) format containing germinal V gene segments from families known to bind DNA (V_H_10) or not (V_H_4) connected to a murine surrogate light chain (SLC), lacking the highly charged unique region (UR), by a hydrophilic peptide linker. We also tested the influence of CDR2 on DNA reactivity by shuffling the CDR2 loop. The scpre-BCRs were expressed in bacteria. V_H_10 bearing scpre-BCR could bind DNA, while scpre-BCR carrying the V_H_4 segment did not. The CDR2 loop shuffling hampered V_H_10 reactivity while displaying a gain-of-function in the nonbinding V_H_4 germline. We modeled the binding sites demonstrating the conservation of a positivity charged pocket in the V_H_10 CDR2 as the possible cross-reactive structural element. We presented evidence of DNA reactivity hardwired in a V gene, suggesting a structural mechanism for innate autoreactivity. Therefore, while autoreactivity to DNA can lead to autoimmunity, efficiently signaling for B cell development is likely a trade-off mechanism leading to the selection of potentially autoreactive repertoires.

## 1. Introduction

Self-antigens are known for their participation in the development and progress of autoimmune diseases such as systemic lupus erythematosus (SLE) [1], rheumatoid arthritis [2] and type 1 diabetes [3] among others. However, the presence of circulating self-antigens itself cannot be attributed to the development of autoimmunity. Several mechanisms act to minimize the effects of potentially autoreactive immune cells through negative selection [4], receptor editing [5] and anergy [6].

Autoreactivity in the immunological system can be observed from the early stages of B cell development. In fact, the majority of the repertoires in the early stages of B cells are potentially autoreactive [7,8]. B-cells precursors are generated in the bone marrow (BM) and necessitate a constant and weak survival signaling to differentiate into circulating B-cells. The constant signaling is mostly originated in the pre-B cell receptor (pre-BCR) expressed on the surface of pre-B cells, ensuring proliferation and survival signals in the bone marrow [9]. Hence, DNA reactive pre-BCRs may benefit from circulating DNA fragments for survival [10].

The pre-BCR contains a rearranged heavy variable domain (V_H_) along with a mouse isotype heavy chain, the μHC. The V_H_ is unique for each clone of pre-B cell clone and is formed in a random rearrangement process that connects three gene segments: variable heavy (V_H_), D, and joining heavy (J_H_), while the V_H_ codes for the CDR1 and 2, D, and J_H_ codes for CDR3, the most variable of the three complementarity determining regions (CDR) [11]. The μHC pairs to the surrogate light chain (SLC) to assemble the pre-BCR. The SLC seems to be an important component for signaling generation interacting with self-antigens through the highly charged unique regions (URs), or with the stromal cells from the bone marrow. The URs are non-Ig domains of the two noncovalent bonded polypeptides, VpreB and λ5, which constitute the SLC [12,13,14]. The SLC is only expressed in a limited temporal stage of B cell development and may be involved in IgH allelic exclusion [15], downregulation of recombinant mechanisms [16], and functionally testing the μH chain, consequently shaping IgH repertoire [17].

The emergence of the rearranged light chain (V_L_) substituting the SLC drives the appearance of the membrane-associated B-cell receptor (BCR). During this developmental stage, the signaling of the newly formed immature B-cell relies on antigenic stimulus for survival and selection [18]. At this point, BCR auto and polyreactivity help to delineate the primary repertoire [19], and polyreactive V gene have been shown to be associated with immunological protection [20]. Nevertheless, the origin and molecular mechanisms involved in autoreactivity and the correlation among individual V genes and self-antigen recognition is still an incomplete scenario.

We previously reported that antibodies harboring a V gene (V_H_) of the mouse V_H_10 family of heavy chain variable gene segments, bind DNA in a disproportional frequency compared to other heavy V gene families [21]. Indeed, recombinant antibodies coded by V_H_10 germline sequences are less dependent on CDR3 to develop anti-DNA binding activity, suggesting that the encoded germline V_H_10 segment itself contains structural elements that facilitate the creation of an anti-DNA paratope [21]. Germline V genes encoded DNA reactivity could help explain the observed part of the B-cell primary repertoire autoreactivity. However, it is unknown if the germline-encoded V_H_10 gene segment is sufficient to encompass a DNA binding site as an innate (germline) property. If it does, this property should be already in any V_H_10 encoded pre-BCR. To test this hypothesis, we constructed a recombinant mouse pre-BCR containing different V_H_ segments and tested DNA binding in vitro. Additionally, we investigated structural features in the V_H_10 germline sequence and located its DNA binding capacity to CDR2.

## 2. Results

### 2.1. Assembling a Recombinant scpre-BCR for Testing V_H_ Intrinsic Binding

**Figure 1:**
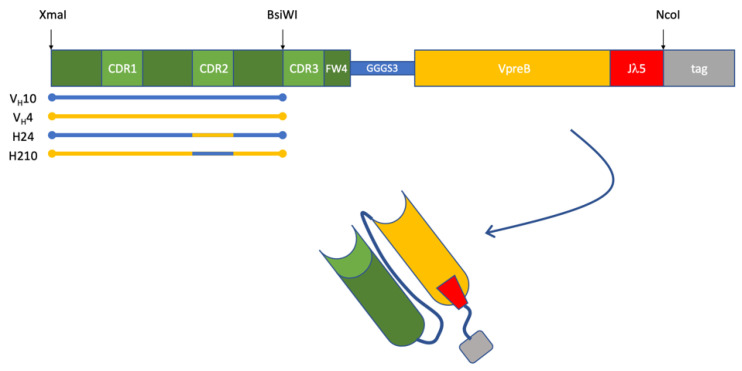
Schematic representation of recombinant scpre-BCR constructs. Selected V genes were put together with a common HCDR3/FW4. A V_L_-like domain was assembled with the mouse VpreB sequence and the λ5 region that functions as an FW4 segment in the native VpreB/λ5 complex. These two variable domains were connected by a (GGGGS)_3_ linker to form a single-chain immunoglobulin pre-BCR domain. Four constructs of these murine scpre-BCR were used in this work, varying their V_H_ counterparts: germinal V_H_10 or V_H_4, and these germinal V segments harboring CDR2 from the other.

To test the germline V_H_10 gene fragments’ intrinsic DNA binding, we designed novel scpre-BCR molecules containing a murine germline V_H_ gene fragment in a pre-BCR context. The recombinant single-chain pre-BCR (scpre-BCR) contained a heavy chain composed of a germline V_H_10, a synthetic CDR3, and a germline FW4. This V_H_ domain combination was based on a previous anti-DNA scFv [21] and was fused through a 15-mer linker to a recombinant fusion of mouse VpreB and λ5, as the J donor (Figure 1). This mouse synthetic VpreBJ construct was based on a human construct from a previous work [22], replacing the human sequences for its mouse homolog for VpreB and λ5 (Supplementary Figure S1). The UR sequences (highly charged unique regions) were removed to reduce nonspecific binding. The loop at the junction of VpreB and λ5 was preserved as described [22].

As a control, three other scpre-BCRs were constructed; the first with a germline V_H_4 family gene fragment, and two others with an artificial sequence derived from the germline V_H_10 gene fragment containing the V_H_4 germline CDR2, and vice-versa (Figure 1). Sequences and alignment are shown in Supplementary Materials. The single-chain constructs also harbored a protein A tag used for affinity purification and immune detection. These recombinant scpre-BCR molecules had molecular weight varying between 36 and 37 kDa and were used to test binding to DNA.

### 2.2. Production and Molecular Characterization of the Recombinant scpre-BCRs

**Figure 2:**
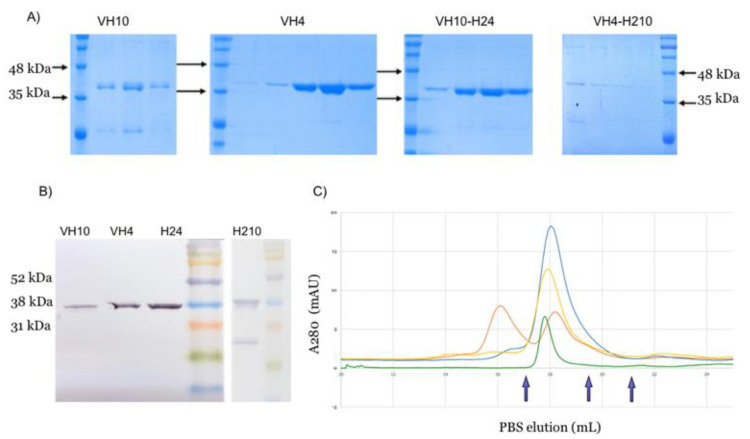
scpre-BCR purification and characterization. (**A**) scpre-BCR V_H_10, V_H_4, V_H_10-H24 and V_H_4-H210 and were produced and fractions obtained from IgG Sepharose affinity purification were analyzed by SDS-PAGE. (**B**) Purified scpre-BCR were analyzed by Western blot. The recombinant proteins were detected by their protein A tag using alkaline phosphatase conjugated rabbit IgG. (**C**) The recombinant proteins samples were also analyzed by size exclusion chromatography (in blue scpre-BCR-V_H_10; in yellow scpre-BCR-V_H_10-H24; in green scpre-BCR-V_H_4-H210 and in orange scpre-BCR-V_H_4). Standards molecular markers are indicated by arrows (left to right: 76 kDa, 29 kDa and 13.7 kDa).

The scpre-BCRs were expressed in sHuffle LysY *E. coli* cells by induction with IPTG at 22 °C. Intracellular soluble fractions were obtained by sonication and affinity purified using rabbit IgG Sepharose column, based on their protein A tag. Eluted fractions were visualized in SDS-PAGE (Figure 2A) and confirmed by Western blot (Figure 2B), Proteins with 37 kDa were dominant on those fractions and correctly immune detected.

The purified proteins were concentrated and analyzed by SEC. SEC profile data showed that scpre-BCR V_H_10, V_H_4-H210 and V_H_10-H24 presented a single peak profile suggesting that these recombinant scpre-BCRs appears as monomers. In contrast, scpre-BCR V_H_4 showed a different profile, with two peaks, indicating that monomeric and dimeric (or an extended monomer) conformations were present for this construct (Figure 2C).

### 2.3. The Germline scpre-BCR-V_H_10 Binds DNA

**Figure 3:**
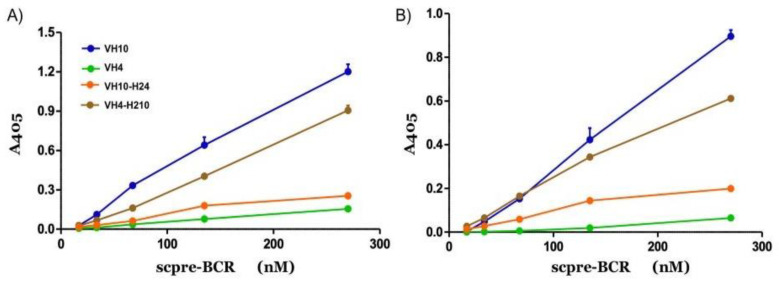
DNA binding activity of scpre-BCRs. The scpre-BCRs were assayed for DNA binding activity by ELISA immunoassay. Plates were coated with either ssDNA (**A**) or dsDNA (**B**), and binding activity of recombinant scpre-BCRs were tested. V_H_10 (blue), V_H_4 (green), V_H_4 germline harboring V_H_10 CDR2 (brown), and V_H_10 germline with CDR2 of V_H_4 (orange) were assayed. Triplicates are shown as mean ± SEM with absorbance at 405 nm plotted against scpre-BCR concentration.

We tested the binding capacity of the four recombinant scpre-BCRs against different forms of DNA molecules through direct ELISA assay. The scpre-BCR containing the germline VH10 gene segment bound more than the other construction to either native or denatured DNA, while the VH4 containing scpreBCR was the worst DNA binder (Figure 3). Interestingly, the exchanged CDR2 scpreBCR-V_H_10-H24 did not show binding activity against the DNA antigens tested, but the scpreBCR-V_H_4-H210, which contains the V_H_10 CDR2 showed an improved binding comparing to scpreBCR-V_H_4.

### 2.4. V_H_10 Germline Sequences Contain DNA Binding Residues in CDR2

**Figure 4:**
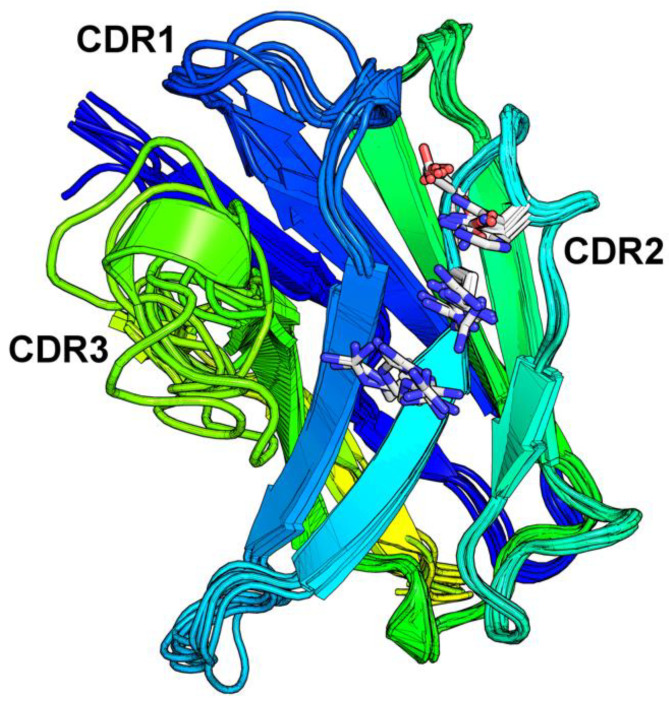
VH10 derived antibodies present the conserved residues Arg50, Arg52, Ser52a and Asn53 which contribute to their anti-DNA reactivity. Superposition of the VH chains of crystal structures of VH10 derived antibodies, PDB codes 4Z8F, 1CBV, 4QWW, 2HKF, 3CXD, 3I2C, 3SGD and 4QNP. The CDR1, CDR2 and CDR3 regions are labeled. The conserved residues Arg50, Arg52, Ser52c and Asn56 are represented as sticks, as is His56 in PDB 3I2C. Numbering follows Kabat convention.

To address the structural role of the V gene segment in the binding of DNA we searched the PDB for V_H_10 containing antibodies. Eight unique entries that used V_H_10 germlines sequences were identified (PDB codes: 4Z8F, 1CBV, 2HKF, 3CXD, 3I2C, 3SGD, 4QNP, 4QWW). Two of them were anti-DNA antibodies (4Z8F and 1CBV). Except for 4ZF8, which appears in the germline configuration, all other V_H_ showed hypermutations leading to residue changes (from two to 11 residues). The structural alignment of the V_H_10 antibodies revealsed a strong superposition (Figure 4) with a reduced RMSD (Supplementary Figure S1). These structures superposed well to each other, and since each antibody had a unique CDR3 loop size and sequence, this region presented higher alignment divergence. The CDR2 was positioned on the side of the molecule revealing a projection of exposed hydrophilic amino acid residues.

The two anti-DNA antibodies bind DNA using heavy chain’s CDR1, 2, and 3. The 1CBV(BV 04-01) structure has low resolution, whereas the 1.75 Å resolution 4Z8F (S1-S15) crystal structure is more reliable, as indicated by the 99th and 96th percentiles in the clashscore and MolProbity scores, respectively [23]. In the former complex a trinucleotide lies along a groove and contacts all three CDRs, while in the second, two oligonucleotides bind simultaneously and shared DNA binding residues in CDR1, 2, and 3 with 1CBV. Since two oligonucleotides molecules bind to 4Z8F, for practical reasons, we may define the paratope as having two binding pockets, one for each oligonucleotide; the first one involving CDR1 and CDR2 contacts and the second involving CDR1 and CDR3 contacts.

**Figure 5:**
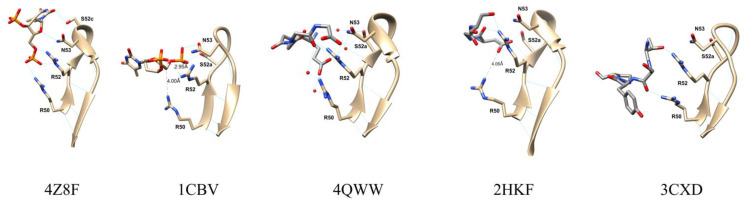
Comparison of antigens contacts in the CDR2 of V_H_10 containing antibodies. The CDR2 of 4Z8F, 1CBV, 4QWW, 2HKF, and 3CXD, from residue 50 to 58 are depicted. Contact residues Arg50, Arg52, Ser52a (or Ser52c for 4Z8F) and Asn53 is shown with side chain and labelled. Water residues (red spheres) are included in the 4QWW model. Antigens contact residues are also in evidence: oligo-dT for the first two and peptide for the others.

In both models, CDR2 seems to contribute with the same germline-encoded residues. Two Arginine residues (50 and 52), in the base of the CDR2, are involved in binding to DNA (Figure 5). In 4Z8F, the Arg^50^ amino group makes a hydrogen bond to phosphate 1 oxygen. In 1CBV this same residue is in Van der Waals (VdW) contact with the sugar backbone. 4Z8F makes two additional hydrogen bonds: Arg^52^ makes a hydrogen bond to another oxygen of the same phosphate group and Asn^53^ makes a hydrogen bond to the first ribose (O4′). The residue Ser^52a^ makes a hydrogen bond to the first phosphate group of the trinucleotide in 1CBV. In 4Z8F Ser^52c^ makes a hydrogen bond to the same O atom. Taken together these five residues, Arg^50^, Arg^52^, Asn^53,^ and either Ser^52a^ or Ser^52c^, are in close contact to antigen in both complexes, making either hydrogen bonds or VdW contacts. Distances to antigen among these residues range from 2.84 to 4.0 Å.

It is interesting to observe that these residues are also involved in complex formation in V_H_10 containing antipeptide antibodies (2HKF, 3CXD, and 4QWW). All four residues are involved in hydrogen bonds to the antigen, except for 4QWW model. In the CDR2 of 4QWW, Ser^52a^ makes no contact but Asn^53^ is in VdW contact (3.87 Å) with the acetylcholinesterase antigen. The model 4QNP, also a V_H_10 antibody, is not symmetrical, and the two Fab binds antigens differently. In this model, the CDR2 residue (Ser^52c^) is the only heavy chain involved in binding to antigen, though the model was not further analyzed. Interestingly, in the 2HFK model that includes water molecules, the four residues contact antigen by direct hydrogen bonds and indirect bonds through four water molecules, suggesting a complex interaction network that traps two carboxyl groups.

**Figure 6:**
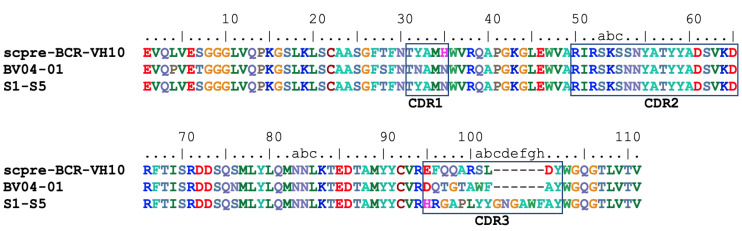
Sequence alignment of V_H_ domain of scpre-BCR-V_H_10 and two anti-DNA antibodies. CDR residues are boxed. Residue numbering and CDR’s follow Kabat definition.

Sequence alignment of the V_H_10 chain used in scpre-BCR with the two anti-DNA antibodies 1CBV [24], 4Z8F [25] shows that the V chain sequences are very similar and share the adjacent arginine residue at position 50 and 52 (Figure 6).Thus, a potential salt-bridge could be formed with DNA’s phosphate groups, allowing the V_H_10 germline chain the ability to bind DNA independently of CDR3 or the V_L_ domain.

### 2.5. A Structural Model of scpreBCR-V_H_10 Supports DNA Intrinsic Binding for DNA

We developed structural models to assess the potential sites for DNA binding in the scpreBCR-V_H_10. Homology modeling was performed based on the 4Z8F structure. Ten best structures were considered for analyses. All models showed a single-chain conventional V_H_-V_L_ pairing. Compared to another pre-BCR structure in PDB (2H32), it showed a similar conformation of the VpreB domain and the J-like strand. The 2H32 structure contained the UR sequences that were exchanged in the VpreBJ synthetic domain for an artificial loop. Nevertheless, in both models, this region preserved the immunoglobulin fold occupying the center of the immunoglobulin barrel in the proximity to the heavy chain CDR3.

**Figure 7:**
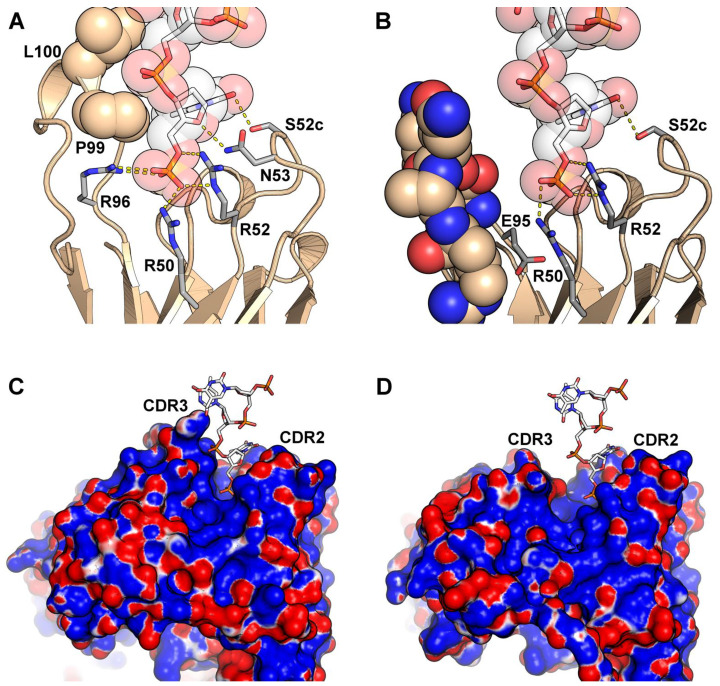
Interactions between anti-DNA antibodies and deoxythymidine oligonucleotides. (**A**) Interactions of labeled residues in both CDR2 (R50, R52, N53 and S52c) and CDR3 (R96, P99 and L100) contribute to deoxythymidine oligonucleotide binding in the crystal structure of the VH10 containing antibody 4Z8F. (**B**) Model of scpre-BCR-VH10 interacting with a deoxythymidine oligonucleotide. The labeled residues R50, R52 and S52c from CDR2 participate in the oligonucleotide binding, while residues from the CDR3 (represented as solid spheres) do not. The CDR3 residue E95 that appears close to R50 is also in evidence. (**C**) Surface electrostatic potential for antibody 4Z8F with the deoxythymidine oligonucleotide represented as sticks. (**D**) Surface electrostatic potential the model of scpre-BCR-VH10 with the deoxythymidine oligonucleotide represented as sticks. In both (**C**,**D**) the CDR2 and CDR3 regions are labeled, and the electrostatic potential was calculated from −3 kT/e to +3 kT/e using APBS, and colored with a gradient from red (negative) to blue (positive).

Interestingly, the model scpreBCR-V_H_10 complexed with a single oligonucleotide suggesting that the VpreBJ domain does not contribute for binding with the antigen (Supplementary Table S1). However, the V_H_ domain showed many contacts and H-bonds, as also observed in the 4Z8F template. The CDR3 residue Glu^95^ seemed to contributes to the binding pocket, appearing beneath the antigen binding pocket (Figure 7).

The majority of CDR2 residues involved in interactions with DNA and peptide antigens (Figure 5) were also observed in the model scpre-BCR. Arg^50^ and Arg^52^ and Ser^52c^ appeared in contact in all models making hydrogen bonds and possibly salt bridges (Supplementary Table S1 and Figure S2). These residues shaped a binding pocket with arginine residues at the bottom. Consequently, the model’s electrostatic surface presented a positively charged path in the CDR2 starting on the paratope floor, running up the CDR2 loop. A comparison of the antibody-DNA complex between 4Z8F and the model scpre-BCR showed that CDR2 dominate the binding in the model scpre-BCR (Figure 7).

## 3. Discussion

We had previously reported that V_H_10 family germline members have an intrinsic preference for DNA binding [21]. Thus, the V gene segment seems to have a major role in this specific binding independent of the CDR3. In this work, we developed an experimental system based on a single-chain molecule to access a germline V_H_10 gene segment’s binding activity as part of a fully reconstitute paratope in a pre-BCR setup. To accomplish this, we needed to consider that the V_H_ gene fragment was only part of the V_H_ domain, which also included a CDR3 and an FW4. Even though the V gene is deterministic to antigen binding [26], it is generally recognized that the heavy chain CDR3 is the most influential region for antigen binding [27]. Thus, we attempted to minimize the CDR3 impact on binding by choosing a sequence that did not favor or hamper DNA binding. We used a synthetic CDR3 previously shown to be neutral for DNA binding, not affecting selection in a phage-display experimental approach, thus mitigating its impact on the recombinant pre-BCR.

To build the recombinant pre-BCR, we chose to assemble it as single-chain antibody fragments. Hence, the V_H_ domain was connected through a flexible linker to a VpreB/λ5 fusion (VpreBJ) domain as a substitute light chain. We constructed a mouse VpreBJ domain based on a human single-domain VpreBJ [22], avoiding the non-immunoglobulin sequences (URs) that project outside of the Ig domain in a tail-like structure. We opted not to include those regions, as they do not contribute to the Ig domain assembly and could bias the binding activity.

Our work focused on the construction of pre-BCR based on the scFv format, a single-chained molecule that emulates a pre-BCR paratope. Antibody fragments such as scFv have been used to investigate the binding properties of several Ig variable domains. The advantage comes from easier and inexpensive expression that can be made in bacterial systems, such as recombinant *E. coli* strains, maintaining antigen-binding properties comparable to the whole molecule [28]. We showed that the scpre-BCR could be purified in soluble form from *E. coli* extracts, using a conventional expression protocol and purification. Interestingly, the scpre-BCR using V_H_4 appeared with two hydrodynamic forms, suggesting that a fraction of it could appear as dimers, or even in an extended state. However, all the other constructions behaved similarly in SEC, compatible with monomer conformation.

The data on the production of scpre-BCR suggest it is an efficient and straightforward scFv-like system for expression and studying individual V_H_ properties. It could also be useful to express antibody fragments that are hard to produce and which binding is dominated by the heavy chain. A similar expression system based on Fab-like pre-BCR was proposed before [29], but here the design was more compact and single-chained, simplifying production and manipulation. Thus, this new kind of molecule is a new acquisition to the toolkit for antibody engineering.

The DNA binding analysis showed that scpre-BCR-V_H_10 bound to different DNA molecule types, in contrast with scpre-BCR-V_H_4 that did not show significant binding activity. However, the insertion of the V_H_10 CDR2 loop (as in scpre-BCR-V_H_4-H210) allowed the V_H_4 germline sequence to bind DNA. Moreover, scpre-BCR-V_H_10-H24, which contains the CDR2 loop derived from the germline VH4, did not show significant binding activity. Thus, the presence of the V_H_10 CDR2 loop seems to be critical for DNA binding in this experimental setup, corroborating the role for the CDR2 in V_H_10 germline sequences. The importance of heavy chain CDR2 in antigen binding has been reported before [25,30].

Our working hypothesis is that the germline V_H_10 gene segment has an intrinsic reactivity to DNA. Therefore, V_H_10 containing antibodies should naturally bind DNA, at least in the initial stages of its ontogeny and, consequently in a V_H_10 containing pre-BCR. Our recombinant soluble pre-BCR bound DNA dependent on the presence of a V_H_10 germline, and especially in the CDR2 shuffled constructs. Taken together, these data suggest a mechanistic role for the germline CDR2 in DNA binding. The participation of CDR2 arginine residues in DNA binding has been reported before [31,32]. Moreover, the structural analysis of the V_H_10-containing antibodies revealed spatial conservation of the CDR2 loop that included a path of hydropathic amino acid residues, with a marked positive charge due to two adjacent arginine residues. In histones, arginine-rich DNA binding proteins, these residues were shown to directly interact with backbone phosphate forming hydrogen bonds and salt bridges [33]. Moreover, clustered arginines were previously implicated in tight binding to phosphate groups [34]. This germline arginine-positive charged path, which appears clearly in the molecular model of the scpre-BCR, suggests a docking pocket, a trap for nucleic acid and other negatively charged molecules.

A dilemma for this hypothesis is that all V_H_10 containing antibodies would naturally be an autoantibody, due to their intrinsic reactivity to autoantigens. Indeed, among V_H_10 sequences in the antibodies database, a large portion are anti-DNA, but not all [21]. Among those not annotated as anti-DNA, there should be a number that are cross-reactive to DNA or other nucleic acid-containing compounds. A clear exact example of this is 4Z8F (mentioned before), an antibacterial lipid A that was later found to be cross-reactive to DNA [25]. Other antibodies could also present, at various degrees, with such a property. Furthermore, V_H_10 antibodies could lose or reduce such an intrinsic property after a negative influence of the rearranged CDR3 [35], or after pairing with an appropriate light chain [30,36]. Additionally, the affinity maturation process may change directly the self-reactive V gene [37].

The data presented here suggest that a germline V_H_10 chain possessed an intrinsic reactivity for DNA. Even though we did not quantitate this affinity, we showed binding to both ss and ds-DNA in the context of an artificial pre-BCR. Furthermore, we also implicate the CDR2 in this autoreactivity. The V_H_10 gene family is poorly used in mouse antibodies, a possible consequence of its autoreactivity. It would be expected that germinal-encoded autoreactivity is deleterious, and eventually eliminated over evolutive time. Autoreactivity seems to be a positively selected trait, and represents a major driver for the B cell repertoire [19,38,39]. Moreover, some V genes are associated with reactivity to commensal microbiota components, such as phospholipid [26], which may be protective to pathobionts [20] and affect the mature B cell repertoire [40,41]. The cross-reactivity shown for the V_H_10 antibody 4Z8F to the phosphate moiety of bacterial Lipid-A [25] suggests that, for V_H_10 antibodies, DNA reactivity brings a premade binding site for other anionic molecules. Therefore, maintaining germline hardwired reactivity for such common antigens seems beneficial, warranting a quick start to immunity in developing animals.

In summary, our results corroborate the hypothesis that a specific murine V gene family can generate anti-DNA antibodies in a pre-BCR context. For that, we developed a novel antibody fragment (scpre-BCR) to test individual V genes. We corroborated the V_H_10 gene segment germline contribution and implicated CDR2 for DNA binding through gain and loss-of function constructs in this scpre-BCR model. Hence, VH10 germline CDR2 codes for a structural arginine trap, suggesting that DNA reactivity can be hard-wired in the germline repertoire.

## 4. Materials and Methods

### 4.1. scpre-BCR Gene Design

Four single-chain pre-BCR gene fragments were designed using germline V_H_ sequences and a mouse surrogate light chain (SLC) engineered based on the previously proposed single domain VpreBJ design concept [22]. Their single domain SLC contained a VpreB sequence fused to a J-like segment of λ5 gene to complete a variable-like VpreBJ domain. The mouse single domain SLC was designed to substitute the human sequences for their mice homologs. The sequences for the murine VpreB region (X05556) and the J region from λ5 (AJ852426) were obtained at the Uniprot database (http://www.uniprot.org accessed on 5 February 2014) (Supplementary Figure S3).

The heavy chain domains were derived from murine germinal sequences of V_H_10 (IGHV10-3*01) and V_H_4 (IGHV4-1*02) families. A third V_H_ domain sequence V_H_10-H24 was built replacing the CDR2 loop (residues 50–58) of the germline V_H_10 for the corresponding sequence in the CDR2 of V_H_4 germline (Kabat numbering and CDR definition). The forth V_H_ construction, named V_H_4-H210, was assembled using the V_H_4 germline inserted with the corresponding V_H_10 CDR2 loop sequence (residues 50–58) (Supplementary Figure S2). All V_H_ gene segments were fused to a common CDR3 and FRW4 based on the mouse J_H_4 [21]. The CDR3 was chosen not to impact binding to DNA. Thus, a single hydrophilic sequence (EFQQARSLDY) was chosen derived from the phage-display selection on oligo dT, that showed a neutral enrichment (selected/unselected ~ 1) either in the V_H_10 or V_H_4 libraries [21]. The single-chain pre-BCR fragment (scpre-BCR) contained one V_H_ and the VpreBJ domains linked by a linker peptide of 15 amino acids residues (GGGGS)_3_.

### 4.2. scpre-BCR Expression Vectors

The scpre-BCR ORF was designed based on *Escherichia coli* highly expressed preferential codons using the Backtranseq program from the EMBOSS package [42]. The coding sequences were chemically synthesized. The scpre-BCR synthetic gene was cloned in the pIg16 expression vector [43] along with a Protein A tag. Initially, the scpre-BCR-V_H_10 plasmid and pIg16 vector was digested with restriction enzymes Xma I and Nco I (New England Biolabs, Ipswich, MA, USA) overnight at 37 °C. Insert and vector were ligated with T4 DNA ligase (Merck, Darmstadt, Germany) incubated overnight at 4 °C, and the transformation was performed by electroporation (Gene Pulser Xcell^TM^ Electroporation System Bio-Rad) using XL1 blue *E. coli* strain. Electroporated samples were cultivated in LB agar plates with 150 μg/mL ampicillin for selection at 37 °C overnight. A recombinant pIg16 vector harboring scpre-BCR-V_H_10 was used for subsequent cloning of V_H_4, V_H_10-H24 and V_H_4-H210 inserts using Xma I (New England Biolabs, Ipswich, MA, USA) and BsiWI (Thermo Fisher Scientific, Waltham, MA, USA) restriction enzymes following manufacturers’ instructions. Recombinant plasmids were checked by Sanger sequencing.

### 4.3. Recombinant Antibody Expression

High-quality Qiagen prepared plasmids were used to transform SHuffle^®^ T7 Express pLysY Competent *E. coli* by heat shock. Transformants were plated in LB agar medium with 150 μg/mL ampicillin and 10 μg/mL chloramphenicol. A preselection step was performed to optimize recombinant protein expression. About twenty random transformant colonies were picked to LB agar medium plates supplemented with 150 μg/mL ampicillin in the presence of either 1% glucose or 1 mM IPTG (isopropyl β-d-1-thiogalactopyranoside, Merck, Darmstadt, Germany). After at least 9 h of incubation at 37 °C colonies that showed a slower growth on IPTG plates compared to that observed on glucose plates were selected for expression. Individual clones were picked and incubated in 5 mL of LB broth supplemented with ampicillin 150 μg/mL overnight at 37 °C and 250 rpm. The following day, 2 mL of the overnight cultivated sample was inoculated in 200 mL LB supplemented with 150 μg/mL ampicillin and incubated at 37 °C, and 250 rpm until an OD of 0.8–1.0 was attained. Cultures were induced with 0.5 mM IPTG overnight at 22 °C and 250 rpm. After induction, cells were centrifuged at 7500 rpm for 10 min and the pellets were stored at −20 °C until use. Recombinant protein production was checked by SDS-PAGE and Western blot (WB). For SDS-PAGE, BLUeye Prestained Protein Ladder (Merk, Darmstadt, Germany) was used, and for the WB assay, we used an Amersham^™^ Protran^®^ 0.2 μm nitrocellulose membrane and Amersham^™^ ECL^™^ Rainbow^™^ Marker Full Range (Merck, Darmstadt,, Germany). After protein transfer, recombinant proteins were probed with a rabbit IgG conjugated with alkaline-phosphatese (AP) (Thermo Fisher Scientific, Waltham, MA, USA).

### 4.4. Recombinant Protein Purification

Pellets from induced cells were resuspended in 15 mL of lysis buffer (75 mM Tris, 300 mM NaCl) and lysed by sonication (Q700/QSonica, Newtown, CT, USA) on ice. Sonication was performed in cycles of 10 s followed by 1 min and 40 s of cooling for 30 cycles (5 min under sonication). The resulting sample was centrifuged at 8000 rpm for 30 min. The soluble fractions were diluted 1:1 in binding buffer solution (10 mM Sodium Phosphate, 500 mM NaCl), filtered using a 0.22 µm filter, and transferred to a 50 mL superloop (Cytiva, Marlborough, MA, USA). Recombinant pre-BCRs were purified by affinity chromatography using a Rabbit IgG-agarose resin (Thermo Fisher Scientific, Waltham, MA, USA). The process was carried out in AKTA Pure System (Cytiva, Marlborough, MA, USA). Binding and elution (0.1 M Glycine, 0.15 M NaCl, pH 2.4) steps were executed following the manufacturers’ guidelines. The fractions were concentrated using 3 kDa Amicon centrifugal filter (Merck, Darmstadt, Germany) and quantified by absorbance (Shimadzu UV1800, Kyoto, Japan).

Selected protein fractions and standards (conalbumin 76 kDa, carbonic anhydrase 29 kDa and ribonuclease A 13.7 kDa) were diluted in PBS and analyzed by size exclusion chromatography (SEC). One mL of each sample was injected in a Superdex™ 200 10/300 GL column (Cytiva, Marlborough, MA, USA) connected to an AKTA Pure System employing a 1 mL/min flow rate.

### 4.5. DNA Binding Assay

The scpre-BCRs were tested on their abilities to bind DNA. Calf thymus DNA (Thermo Fisher Scientific, Waltham, MA, USA) was used to prepare samples of dsDNA (20 μg/mL) and ssDNA (20 μg/mL) to coat the ELISA plates (Nunc, Maxisorp 96 well, Thermo Fisher Scientific, Waltham, MA, USA). The ssDNA from calf thymus was obtained through boiling for 20 min and then promptly transferring it to ice immediately before its use for coating the plate. Before coating, the plates for ds and ssDNA assay were irradiated with UV light for 25 min to optimize DNA adsorption to the plate. Coating of ds and ssDNA were performed overnight at 4 °C. A serial dilution (dilution ratio 1:1) of the scpre-BCRs on the coated plates was conducted using an initial concentration of 10 μg/mL with resulting concentrations of 5, 2.5, 1.25, and 0.625 μg/mL. The amount of protein added was normalized by scanning densitometry of SDS-PAGE samples. The concentration of each sample was normalized considering the area of the most abundant, expected sized, band. To detect the binding activity the protein A tag was probed with rabbit IgG conjugated with alkaline phosphatase (AP) (Thermo Fisher Scientific, Waltham, MA, USA). p-Nitro-Phenyl-Phosphate (PNPP) was used as chromogenic substrate at 1mg/mL (Thermo Fisher Scientific, Waltham, MA, USA). The assays were performed in triplicates. All incubation steps lasted for 1 h with 3× PBST washes between them.

### 4.6. Structural Evaluation of V_H_10 Antibodies

The structure of V_H_10 containing antibodies was accessed from the Protein Data Bank (www.rcsb.org accessed on 1 May 2020) after their identification using the Blast program at NCBI (blast.ncbi.nlm.nih.gov accessed on 30 April 2020). The mouse germline V_H_10 sequence (IGHV10-3*01) was blasted to the PDB dataset. The best scoring heavy chain was checked for germline identification using IgBlast (https://www.ncbi.nlm.nih.gov/igblast/ accessed on 15 May 2020). We considered for analyses the best scoring PDB entries identified by IgBlast as composed by a mouse V_H_10 germline sequence. Structures were analyzed using Chimera [44]. Distances, contacts, and hydrogen bonds were retrieved with Cocomaps [45]. Structural superposition and graphics were generated with Chimera. The numbering followed Kabat’s definition and was assigned using Abnum (http://www.bioinf.org.uk/abs/abnum/ accessed on 19 January 2021).

### 4.7. Modeling of the scpre-BCR-V_H_10

The deposited sequence, structure model and electron density map coefficients of the antigen-binding fragments of the S1–15 anti-lipid A monoclonal antibody in complex with a DNA oligonucleotide 5P-TTTTT-3P were retrieved from the Protein Data Bank (PDB code 4Z8F). The structure model and electron density were analyzed using the program COOT [46]. The water molecules were removed from the crystallographic model, and this model was used as a template in the subsequent molecular modelling steps. The sequence of the scpre-BCR-V_H_10 was manually aligned to the 4Z8F sequence. The program MODELLER 9.24 [47] was employed to generate several comparative molecular models of the scpre-BCR-V_H_10. The stereochemistry of the models was evaluated using the Molprobity server [23].

The I-TASSER server [48] was also employed to produce homology models of the scpre-BCR-V_H_10. I-TASSER modeling was guided by tailored restraints based on the 4Z8F deposited crystal structure. Additionally, I-TASSER used LOMETS to identify several deposited crystal structures that were used as threading templates. The quality of each model was accessed by the C-score and the RMSD values.

## Supplementary Materials

The following are available online at https://www.mdpi.com/article/10.3390/ijms22094541/s1, Figure S1: Scheme of scpre-BCR recombinant protein, Figure S2: Alignment of recombinant V_H_ gene segment produced as scpre-BCR, Figure S3: Structural alignment of eight VH10 containing PDB models, Table S1: WdV contacts and H-Bonds in model scpre-BCR-VH10.

## Abbreviations

AP	Alkaline Phosphatase
BCR	B Cell Receptor
BM	Bone marrow
CDR	Complementarity Determining Region
dsDNA	Double strand DNA
ssDNA	Single strand DNA
ELISA	Enzyme-Linked Immunosorbent Assay
FW	Framework region of variable domains
IgH	Immunoglobulin heavy chain
IPTG	Isopropyl β-d-1-thiogalactopyranoside
LB	Luria-Bertani broth
PBST	Phosphate Buffered Saline added by tween 20 (0.5% *v*/*v*)
PDB	Protein Data Bank
PNPP	ρ-Nitro-Phenyl-Phosphate
RMSD	Root-Mean-Square Deviation
scpre-BCR	Single chain pre-B cell receptor
SEC	Size Exclusion Chromatography
SLC	Surrogate light chain
UR	Highly charged unique regions of the SLC
VDW	Van der Waals Interactions
VH	Heavy chain variable domain
VL	Light chain variable domain
