# Supplementary material for: A Germline-Encoded Structural Arginine Trap Underlies the Anti-DNA Reactivity of a Murine V Gene Segment
Source: Int J Mol Sci. 2021 Apr 26;22(9):4541. doi: 10.3390/ijms22094541 (PMC8123574; doi:10.3390/ijms22094541)
Supplement: Supplementary file 1 [file ijms-22-04541-s001.zip › ijms-1136630-supplementary.pdf]

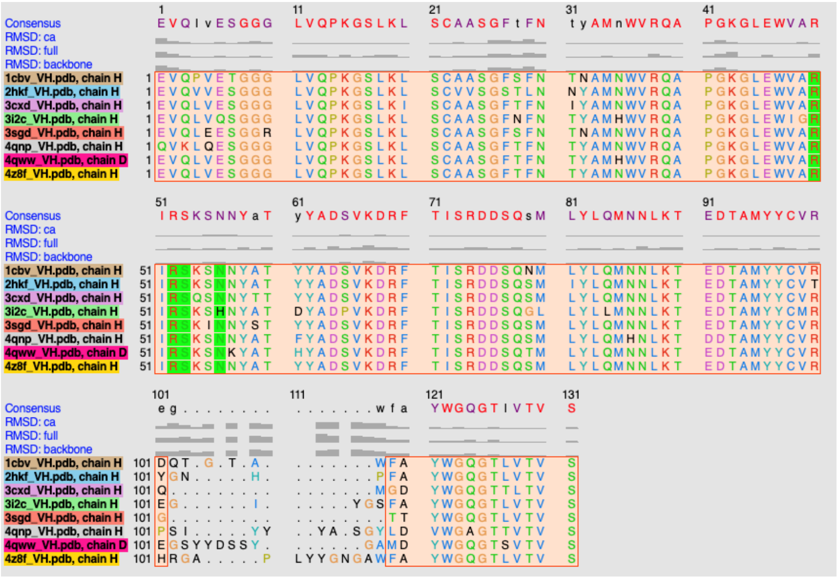

Figure S1. Structural alignment of eight VH10 containing PDB models. CDR2 residues that are depicted in Figure 4 is marked in green. Alignment was performed with Chimera for the V_H segment (residues 1–100).

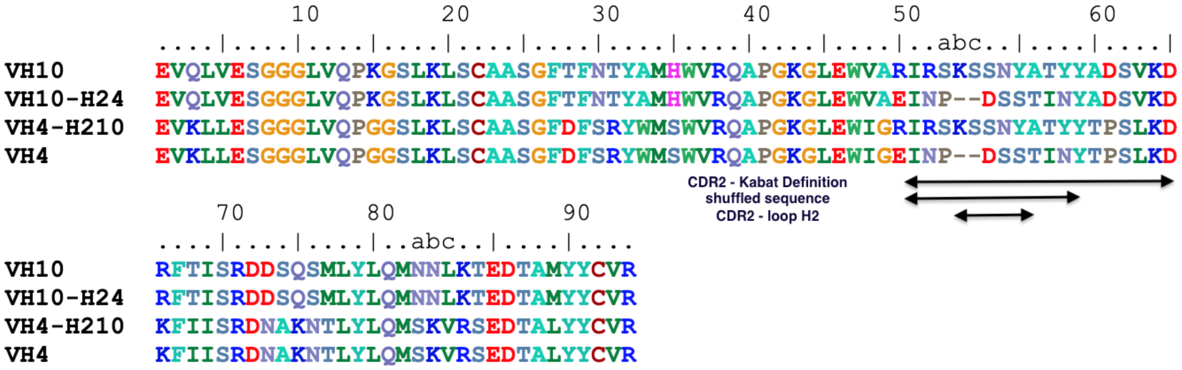

Figure S2. Alignment of recombinant V_h gene segment produced as scpre-BCR. VH10, VH4 and the two CDR2 shuffled variants. VH10-H24 correspond to the VH10 germline containing the CDR2 loop region of VH4 germline. VH4-H210 correspond to the VH4 germline containing the CDR2 loop region of VH10 germline. CDR2 Kabat definition, shuffled region and H2 loop is marked with double headed arrows.

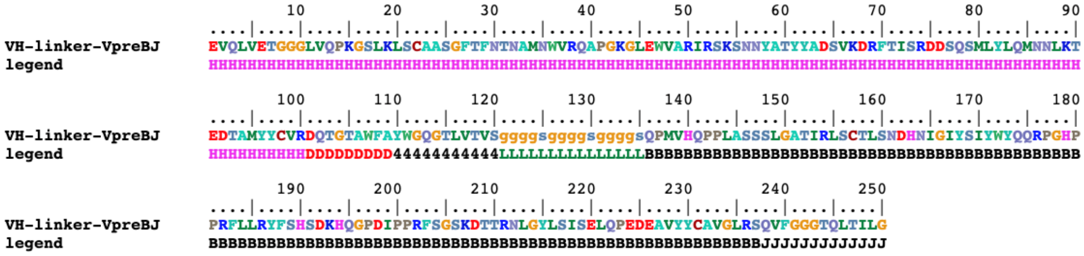

H – Germline heavy chain (V_H10)
D – Synthetic D segment
4 – J_H4 gene segment
L- linker sequence
B- VpreB
J – λ5 J-like FW4

Figure S3. Scheme of scpre-BCR recombinant protein.

Table 1. WdV contacts and H-Bonds in model scpre-BCR-VH10. The number of times a residue makes contacts or bonds among the best 10 models is tabulated. Residues <5.0 Å apart are considered counted as WdV contact residues. H Bonds and distances are calculated with Chimera.

Residue	Contact	H-Bond	Location
T31	10	0	CDR1
A33	10	0	
R50	10	4	CDR2
R52	10	5	
S52c	10	6	
S53	3	0	
E95	8	0	CDR3
Q98	1	0	
R100	3	0	
R237	1	0	VpreB
